# Early-Age Manifestation of Singleton Merten Syndrome With Systemic Lupus Erythematosus Features: A Case Report

**DOI:** 10.7759/cureus.25244

**Published:** 2022-05-23

**Authors:** Yazeed M Alzahrani, Ahmad A Alamoudi, Nojoud K Nahar, Rawia F Albar

**Affiliations:** 1 College of Medicine, King Saud Bin Abdulaziz University for Health Sciences, Jeddah, SAU; 2 Pediatrics, King Abdulaziz Medical City, Jeddah, SAU

**Keywords:** joints calcification, persistent hypertension, congenital aortic calcification, systematic lupus erythematoses, singleton merten syndrome

## Abstract

Singleton Merten syndrome (SMS) is one of the rarest multisystem genetic disorders that had been recognized in only a few cases. Patients who have this syndrome often present with calcification of the aorta and heart valves, dental dysplasia, joint calcification, distinct facial features, and growth and developmental delay. Other physical findings usually associated with SMS may include glaucoma, skeletal abnormalities including tendon rupture, muscle weakness, and arthropathy. In individuals with SMS, autoimmune diseases like psoriasis and systemic lupus erythematosus (SLE) can occur. In this case, we report a pre-term baby girl that developed congenital aortic calcification, renal hypertension, dental anomalies, multiple joint calcifications, atypical facial features, mild mental retardation, and developmental delay. At 17 years, the patient developed SLE based on positive antinuclear antibody (ANA) with clinical and immunological features like fever, malar rash, pericardial effusion, proteinuria, high ANA concentration, high anti-double-stranded DNA, low C4 complement, and presence of anti-Smith antibodies.

## Introduction

Singleton Merten syndrome (SMS) is known as an autosomal-dominant disorder that is characterized mainly by aortic calcification, dental malformation, and skeletal abnormalities which manifest in early life [1]. SMS is a type I interferonopathy that can present also with other clinical findings including muscle weakness, fever of unknown origin, and short stature due to the growth lag. Osteoporosis and progressive loss of bone minerals with multiple limb deformities are common findings of SMS [2]. The pathophysiology of aberrant calcification is yet uncertain; however, it is thought to be connected to a gain-of-function mutation in factor inhibiting hypoxia-inducible factor 1 (IFIH1) [3]. The IFIH1 gene makes melanoma differentiation-associated gene 5 (MDA5), which mainly plays a role in the immunity of certain viral diseases. IFIH1 gain-of-function mutations lead to the inappropriate interferon (IFN) response due to excessive inflammatory process, which leads to extensive organ damage and calcification [4]. In this case, a young female patient presented with SMS and in addition developed systemic lupus erythematosus (SLE) at a later age. 

## Case presentation

This is a case of a baby girl that was born prematurely at 32 weeks and delivered by emergency cesarean section for fetal distress and flat cardiotocograph tracing. The baby was born flat with an Apgar score of 6 at 5 minutes. She was intubated, resuscitated, connected to mechanical ventilation, and kept in the neonatal intensive care unit for almost two months. Her birth weight was 1.9 kg, head circumference 32 cm, and length 40 cm, which is appropriate for gestational age. Initial vital signs and clinical examination were normal. Mother is gravida 2 para 0 + 1. Parents are first-degree cousins. The first pregnancy ended in miscarriage at six weeks subsequently and the mother failed to conceive for four years, after which she conceived spontaneously. At 15 hours of age, her blood pressure increased to 104/70 and repeated cardiac examination revealed a significant heart murmur grade 4, harsh and systolic, and was heard all over the pericardium. Chest X-ray revealed cardiomegaly and the possibility of patent ductus arteriosus due to the heart examination which revealed the murmur. The patient was managed by fluid restriction, increased positive end-expiratory pressure, and Lasix, and despite this, her blood pressure remained high. Then, she received two doses of hydralazine which had a temporary effect. However, after a few hours, the patient looked worse, was poorly perfused and mottled, and developed hepatomegaly and abdominal distension. She had tachycardia of 160/min and was diagnosed as having congenital heart failure. Echocardiography showed thick left ventricular wall thickness, moderate mitral regurgitation, left-to-right shunt across patent foramen ovale, and patent ductus arteriosus.

**Figure 1:**
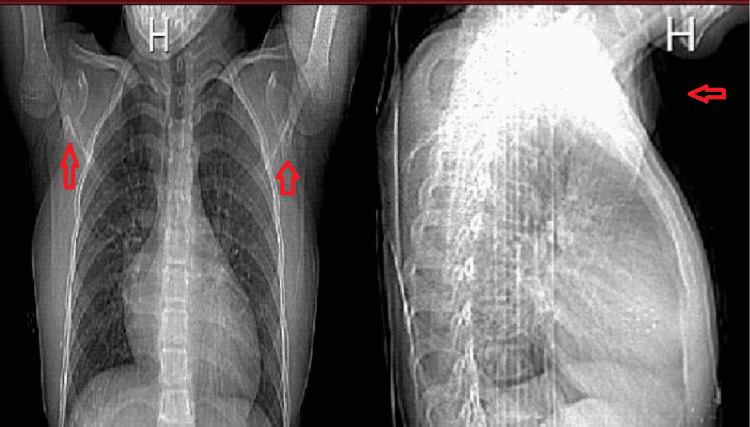
X-rays showing multiple calcifications These X-rays show multiple calcifications on the upper extremity, especially in the shoulders. They also show cardiomegaly in the patient due to the heart abnormality.

At nine weeks of age, the patient was diagnosed with congenital heart failure with an unknown cause and was managed with digoxin, diuretics, and ventilation. Hypertension was difficult to control and her blood pressure was 122/84 and on three antihypertensive drugs which are nifedipine, captopril, and hydrochlorothiazide with borderline control. A few days later, endotracheal tube secretion grew *Pseudomonas* as well as the blood culture and she was managed with IV ceftazidime and vancomycin. The swelling was noted on the right forearm, which resembled an abscess, and was aspirated and sent for culture which also showed *Pseudomonas*. Multiple joint calcifications that were noticed through X-ray raised the possibility of a diagnosis of infantile idiopathic intra-arterial calcification (Figure 1). She was then discharged and was given multiple antihypertensive drugs, which are nifedipine, captopril, and hydrochlorothiazide, and scheduled for follow-ups.

**Figure 2:**
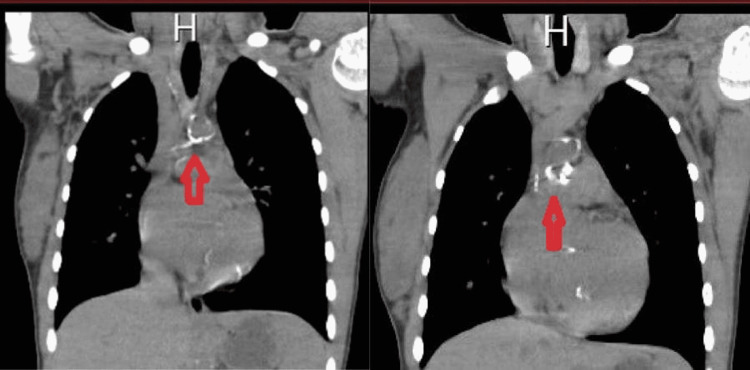
CT scan of the heart These CT scans of the heart revealed calcification of the descending aorta. CT, computerized tomography.

At 10 months she presented with a puffy eye and high readings of blood pressure, 130/85, were noticed, so she was admitted to control hypertension. On examination she was well, pink in room air with an obvious squint on the right eye that was later corrected by surgery. She was not pale or jaundiced. Chest showed bilateral equal air entry with no added sound. On cardiovascular examination first and second heart sounds were loud with no signs of murmur. The abdomen was lax and had no organomegaly and masses. There was restricted joint movement in the upper limb and can not abduct more than 120 degrees, whereas lower limbs were normal. Dimercaprol succinic acid (DMSA) scan showed that the left kidney had a good uptake of 71% and showed evidence of compensatory hypertrophy, while the right kidney showed a poor uptake of 29% and it was small. Ultrasound of the kidney showed the size of the right kidney to be 1.9 × 6 cm and the left kidney to be 2.6 × 2.4 cm. Both showed multiple cysts of different sizes and the smallest was 2 mm while the largest was 90 mm in diameter. The patient was diagnosed to have bilateral infantile polycystic kidney disease and renal resistant hypertension. On urinalysis, it was found that the urine protein of 30 g\L and creatinine was 49 mg/dL. At three years of age, upon follow-up, the patient was noted to have mild mental retardation, low IQ, and delayed speech and development. Moreover, the patient was noticed to be short for her age and diagnosed with rickets. The patient was noted to have caries on the teeth and malformation, so the patient was referred for full mouth rehabilitation involving multiple extraction and restorations. Also, the patient was admitted for bilateral genu valgus deformity, and was referred for correction surgery. Later, the patient was followed up and recurrent right genu valgum deformity was noticed and sent for corrective osteotomy. At 11 years of age, she was complaining of recurrent tonsillar attacks more than seven per year for several years, so an adenotonsillectomy was done to relieve the patient’s pain. Some dysmorphic features like a long face, prominent nose, epicanthic fold, and short stature were suspected to rule out Turner syndrome which was ruled out by chromosome analysis which revealed an apparently normal female karyotype. When 12 years old the patient was not compliant with the medications and was admitted to the intensive care unit because of high blood pressure of 200\40 and was stabilized with multiple hypertensive medications and then discharged. At 13 years of age upon follow-up with her nephrologists, they noted normal kidney imaging and function and there was no explanation for resistant hypertension. Recently, her mother had a pregnancy, and upon follow-up, the fetus was diagnosed with idiopathic arterial calcification and the fetus died in utero. Based on this finding the patient underwent computerized tomography (CT) which confirmed the presence of significant calcification on the descending aorta which was not diagnosed before (Figure 2).

SMS was diagnosed based on the clinical findings of this patient. Other tests that help to confirm the diagnosis were done including an X-ray which showed the presence of joint calcification, especially in the upper and lower extremities (Figure 1). The identification of calcification in the aorta in the CT (Figure 2), together with the other findings listed above, strongly suggests that SMS is present. In addition, whole-exome sequencing was done at 16 years of age, which confirmed the diagnosis of generalized arterial calcification of infancy (GACI) type 1 caused by pathogenic mutations in the ectonucleotide pyrophosphatase/phosphodiesterase 1 (ENPP1) gene. When 17 years old, the patient developed SLE. The diagnosis was established based on the Systemic Lupus International Collaborating Clinics (SLICC) criteria and the patient fulfilled it with evident positive antinuclear antibody (ANA), anti-double-stranded DNA (anti-dsDNA), anti-Smith (anti-SM) antibodies, and low C4 complement (Table 1). In addition, an echocardiogram was done and a large pericardial effusion was noted with severe concentric left ventricular hypertrophy. Also, laboratory testing showed high 24-hour urinary protein content (Table 1).

**Table 1:** Laboratory findings. MCV, mean corpuscular volume; MCH, mean corpuscular hemoglobin; RDW, red cell distribution width; ESR, erythrocyte sedimentation rate; PTT, partial thromboplastin time; eGFR, estimated glomerular filtration rate; LDH, lactate dehydrogenase; BUN, blood urea nitrogen; ANA, antinuclear antibody; anti-dsDNA, anti-double stranded DNA; anti-SM, anti-Smith.

Variables	Results	Reference values
Blood routine		
Hemoglobin (g/dL)	9.4	11.6-15
Hematocrit (%)	29.1	35.5-44.9
White cell count (×10^9^/L)	6.9	3.5-9.5
Platelet count (×10^9^/L)	297	125-350
MCV (fL)	64.8	80-100
MCH (pg)	21.5	27.5-33.2
RDW (%)	16.3	12.2-16.1
ESR (mm/h)	34	1-20
PTT (s)	47	25-35
Electrolyte		
Potassium (mmol/L)	3.1	3.5-5.3
Magnesium (mmol/L)	0.69	0.85-1.10
Calcium (mmol/L)	2.25	2.2-2.7
Sodium (mmol/L)	137	137-147
Chloride (mmol/L)	103	99-100
Blood biochemical		
eGFR (JD) (mL/min/1.73 m^2^)	44	>60
Uric acid (umol/L)	721	148-422
Alkaline phosphate (IU/L)	261	44-147
LDH (mmol/L)	266	0.5-2.2
Creatinine (Umol/L)	114	53-97.2
Protein urine (mg/day)	605	<150
BUN (mmol/L)	14.8	2.1-8.5
Antibodies		
ANA	Positive	Negative
Anti-dsDNA (IU/mL)	Positive	Negative
Anti-SM	Positive	Negative
C4 complement (g/L)	0.09	0.16-0.48

Besides having SMS and SLE, the patient is currently diagnosed with severe uncontrolled hypertension and is on four antihypertensives with very low diastolic blood pressure due to loss of aortic elasticity because of extensive calcification and narrowed abdominal aorta, polycystic kidney disease, mild renal impairment, mitral annular calcification, and gout. She is in stable condition on cholecalciferol (D3) tablet (2000 units oral for 360 days), spironolactone, furosemide, carvedilol, indapamide, amlodipine, isosorbide, aspirin, and prednisolone for heart disease, renal disease, hypertension, and SLE. The patient is compliant with the medications and in a stable condition with regular follow-ups with cardiology, nephrology, and rheumatology to check for any development regarding intra-arterial calcification, SLE, and renal disease. 

## Discussion

SMS is one of the rare genetic diseases, with only a few cases documented in the literature until now [5,6]. The exact reason why a single gene is linked to so many different autoimmune diseases with varied phenotypes is yet unclear. One hypothesis is that MDA5 becomes responsive to distinct endogenous double-stranded RNAs (dsRNAs) due to conformational changes caused by different amino acid modifications [6,7]. Diagnosis is significant in type I IFN-driven disorders, because targeted anti-IFN treatments, such as Janus kinase-signal transducer and activator of transcription (JAK-STAT) inhibition and anti-IFN receptor antibodies, provide tailored treatment aimed at the underlying genetic etiology [8,9].

The main clinical manifestations of SMS are aortic calcification, abnormal skeletal osteogenesis of the extremities, and dental malformation [5,6], which are all common features of this syndrome and were seen in our patient. However, it can also involve less common features including glaucoma, psoriasis, tendinitis, and osteoporosis [6,10]. In our case, the patient developed resistant hypertension of unknown cause, rickets, and squint as non-common features of SMS.

This case presented with aortic calcification, dental malformation, and joint calcification, and these characteristics are part of the diagnosis of SMS. A genetic test was done and it confirmed the diagnosis of GACI type 1. The interpretation of the report was that GACI is caused by pathogenic mutations in the ectonucleotide pyrophosphatase/phosphodiesterase 1 (ENPP1) gene. Although adult survival has been reported in a few cases, GACI is typically fatal within the first six months of life due to myocardial ischemia leading to refractory heart failure. Additionally, facial features might be a distinguishing mark of SMS patients with IF1H1 gene gain-of-function mutations. It has been reported that SMS patients can present with specific facial features, such as a broad forehead, high anterior hairline, thin upper vermilion, and smooth philtrum [10]. Likewise, our case presented atypical facial features. 

Another noteworthy condition is that SMS can be associated with many autoimmune diseases, including diabetes, psoriasis, autoimmune thyroid disease, and SLE [11-14]. In our case, SLE was diagnosed based on positive ANA and clinical features which are fever, malar rash, pericardial effusion, and proteinuria. For the immunologic features, there are high ANA concentration, high anti-dsDNA, low C4 complement, and presence of anti-SM antibodies.

Cases with SMS and IFIH1 gain-of-function mutations often develop thrombocytopenia. To our knowledge, pancytopenia is an uncommon feature in cases with these mutations. The actual mechanism causing pancytopenia as a result of the IFIH1 gain-of-function mutation remains unclear [15]. The patient’s leukocyte and platelet counts were normal. However, hemoglobin level was below the normal range. Besides these landmarks of the syndrome, our patient had a low IQ score of 64 and mild mental retardation. In addition, our patient had a developmental delay at a young age and her height is below average, which is a common finding in individuals with SMS.

## Conclusions

SMS is one of the rarest genetic diseases, and in this case, we highlight a patient with this syndrome that developed since birth and all the abnormalities that we encountered. Due to its rarity, we encourage making a genetic diagnosis and understanding the whole phenotypic spectrum in these families, which is significant for customized management and screening, as well as providing genetic testing including prenatal testing to at-risk relatives. In addition, since SLE may develop in individuals with SMS, early measures are advised.
